# Inefficient maturation of disease-linked mutant forms of the KCC2 potassium-chloride cotransporter correlates with predicted pathogenicity

**DOI:** 10.1016/j.jbc.2025.108399

**Published:** 2025-03-10

**Authors:** Morgan Kok, Ishika Singh, Elias Aizenman, Jeffrey L. Brodsky

**Affiliations:** 1 Department of Biological Sciences, University of Pittsburgh, Pittsburgh, Pennsylvania, USA; 2 Department of Neurobiology, University of Pittsburgh School of Medicine, Pittsburgh, Pennsylvania, USA

**Keywords:** potassium transport, glycoprotein biosynthesis, endoplasmic-reticulum-associated protein degradation (ERAD), computational biology, epilepsy

## Abstract

The potassium-chloride cotransporter 2 (KCC2) is required for neuronal development, and KCC2 dysregulation is implicated in several neurodevelopmental disorders, including schizophrenia, autism, and epilepsy. A dozen mutations in the KCC2-encoding gene, *SLC12A5*, are associated with these disorders, but few are fully characterized. To this end, we examined KCC2 biogenesis in a HEK293 cell model. While most of the examined disease-associated mutants matured efficiently, the L403P mutant was unable to traffic to the Golgi. Two other mutants, A191V and R857L, exhibited more subtle defects in maturation. Cell surface biotinylation assays showed that these mutants were also depleted from the cell surface. Another disease-associated variant, R952H, acquired Golgi-associated glycans yet was significantly depleted from the plasma membrane, consistent with loss of a plasma membrane–stabilizing phosphorylation site. To determine whether the ability of KCC2 to mature to the Golgi could be predicted, we employed a computational pathogenicity program, Rhapsody, which was shown in past work to predict endoplasmic reticulum–associated degradation-targeting of an unrelated ion channel. We discovered that the Rhapsody pathogenicity score correlated with relative defects in KCC2 maturation, and the algorithm outperformed two other commonly used programs. These data demonstrate the efficacy of a bioinformatic tool to predict the efficiency of KCC2 biogenesis. We also propose that Rhapsody can be used to develop hypotheses on defects associated with other disease-associated *SLC12A5* alleles as they are identified.

Neurodevelopmental disorders initiate in early brain development at a point at which synaptogenesis is critical and are characterized by chronic deficits in memory, cognition, behavior, and/or motor skills (1). Because synaptogenesis relies upon the activity of neuronal ion transporters, dysregulated activity due to ion transporter malfunction—which can be caused by the presence of deleterious mutations—disrupts neurodevelopment, leading to altered neuronal function (2, 3). For example, a dozen mutations in the neuron-specific K^+^/Cl^-^ cotransporter 2 (KCC2) are linked to epilepsy, autism spectrum disorder (ASD), and schizophrenia (4). Current therapies for these diseases are limited by severe side effects and a lack of specificity for the molecular targets underlying the disease (5, 6). Among the KCC2 mutant alleles associated with these diseases, only a few are characterized, and some mutations in the gene-encoding KCC2 have been identified in more than one disorder (7, 8, 9). As such, a deeper molecular characterization of disease-associated alleles in the gene-encoding KCC2 represents the first step toward the development of target-specific therapies.

The neuron-specific KCC2 protein is the product of the *SLC12A5* gene and is the major chloride extruder in the adult brain, yet KCC2 is perhaps best known for its role in regulating γ-aminobutyric acid (GABA) signaling (10). During brain development, GABA undergoes a pivotal switch from mediating excitatory to inhibitory neurotransmission (11). This event is facilitated by a concomitant developmental increase in KCC2 expression (12, 13). In early prenatal brain development, KCC2 expression is low, and high intracellular chloride concentrations ([Cl^-^]_i_) trigger chloride exit through the GABA_A_ receptor, thus depolarizing the plasma membrane and initiating an excitatory response. In contrast, late development (starting at postnatal week 2) is characterized by elevated levels of KCC2, resulting in increased chloride efflux and reduced [Cl^-^]_i_. As a result, chloride enters neurons through the GABA_A_ receptor, and the membrane is hyperpolarized, thereby producing an inhibitory signal (14). Previous research established that KCC2 is indispensable for the transition in GABA signaling, and a reduction in KCC2 function *via* knockdown studies in mice resulted in neuronal hyperexcitability and seizures (15, 16, 17). Indeed, impaired GABAergic inhibition and neuronal hyperexcitability have similarly been implicated in multiple neurodevelopment disorders, including epilepsy, ASD, and schizophrenia (18, 19, 20, 21). While there are FDA-approved drugs that ameliorate disease symptoms by targeting the GABA pathway (22), they are not allele-specific and—as noted above—may result in serious side effects.

As a member of the solute carrier 12 (SLC12) family of cation-chloride cotransporters, KCC2 is predicted to fold into an interwoven dimer with each monomer containing 12 transmembrane domains, intracellular N- and C-termini, and a large extracellular loop (23, 24, 25). Due to alternative promoter usage, two isoforms (KCC2a and KCC2b) of the transporter exist, which differ in the first 40 N-terminal amino acids (26). While the isoforms exhibit similar activities, the levels of KCC2b increase throughout brain development while KCC2a levels remain relatively low in whole brain lysates (13, 27). Nevertheless, the extracellular loop in both KCC2 isoforms contains six sites for N-linked glycosylation (N283, N291, N310, N328, N338, and N339), and previous work has shown that the N-terminal domain is generally responsible for KCC2 delivery to the plasma membrane, whereas the C-terminal domain is required for ion transport (28, 29, 30). In addition, multiple phosphorylation sites have been identified throughout the protein, with some located in the C-terminal domain. These play a role in KCC2 stability and activity at the cell surface (2, 4). For example, phosphorylation of S940 decreases KCC2 internalization (31), but phosphorylation of Y903 and/or Y1087 promote internalization (32). In contrast, phosphorylation of T906 and T1007 has an inhibitory effect on KCC2 ion transport (33, 34).

Of the 12 disease-associated amino acid substitutions identified in KCC2, 11 are linked to epilepsy (A191V, L288H, W318S, S323P, S376L, L403P, M415V, G528D, R857L, R952H, and R1049C), three are linked to ASD (R952H, R1048W, and R1049C), and one is linked to schizophrenia (R952H) (Fig. 1*A*) (2, 4). R952H and R1049C have been identified in more than one neurodevelopmental disorder (7, 8, 9). Patch-clamp analyses of L288H, L403P, M415V, and G528D in transfected HEK293 cells uncovered significantly reduced Cl^-^ extrusion relative to the WT transporter (35, 36). Other patch-clamp experiments in Neuro2a cells transfected with message encoding the R952H and R1049C mutant alleles also indicated reduced Cl^-^ extrusion (8). Moreover, cell surface biotinylation assays in transfected HEK293 cells showed decreased levels of L288H, G528D, and L403P at the plasma membrane, while A191V and M415V protein levels were unchanged (35, 36). These data suggest that some KCC2 variants are stable and traffic properly through the secretory pathway, but they exhibit defects in ion transport. In contrast, fluorescence-based detection assays in transfected hippocampal neurons established that R952H—but not R1049C—exhibit reduced cell surface expression (8). Interestingly, experiments in HEK293 cells also established that phosphorylation of S940 in the R952H and R1049C KCC2 mutants was reduced (9). Therefore, other KCC2 mutations appear to disrupt the native conformation or alter critical posttranslational modifications, which likely contributes to disease pathogenesis. Nevertheless, several disease-associated mutants (*i.e.*, W318S, S376L, R857L, and R1048W) are uncharacterized (7, 36, 37). With a surge in the discovery of putative disease-associated mutations—as a result of expanded human genome databases—it is becoming increasingly critical to predict the disease severity and molecular consequences of mutations in KCC2 and other transporters and ion channels.

**Figure 1:**
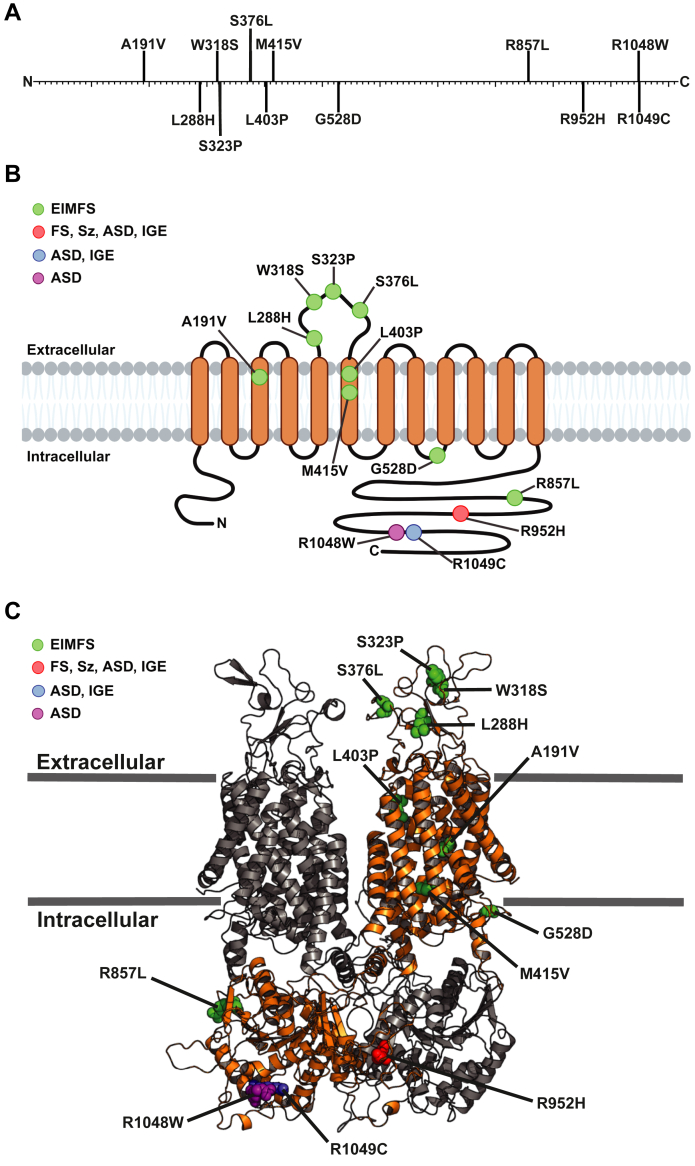
**Multiple disease-associated mutations have been identified in KCC2.***A*, linear representation of the KCC2 amino acid sequence. Disease-associated KCC2 mutations are mapped to their respective locations. *B*, topological model of monomeric KCC2 located at the plasma membrane. Mutations are labeled in their general locations and color coordinated according to their associated disease(s). Diseases include epilepsy of infancy with migrating focal seizures (EIMFS), febrile seizures (FS), schizophrenia (Sz), autism spectrum disorder (ASD), and idiopathic generalized epilepsy (IGE). Image created with Biorender.com. *C*, 3D homology model of dimeric KCC2 on the plasma membrane. To distinguish each subunit, monomers are shown in *gray* and *orange*. Mutations are mapped on the *orange* subunit and color coordinated with their associated disease(s) as described in (*B*). This KCC2 homology model was obtained from SWISS-MODEL and created using the KCC3 cryo-EM structure (PDB: 6Y5R). KCC2 and KCC3 are 77% sequence identical. Image rendered using PyMOL (version 2.2.3).

To this end, we compared the maturation, stability, and cell surface residence of six partially characterized or uncharacterized disease-associated KCC2 mutants in both transient and stable HEK293 expression systems. While some of the mutant forms of KCC2 matured and were transported to the plasma membrane in a WT-like fashion, others exhibited dramatic maturation defects such that their delivery to the Golgi *via* the canonical secretory pathway was interrupted. Yet, the proteins were only modestly unstable, suggesting that folding defects were sufficiently subtle to limit delivery to the ER associated degradation (ERAD) pathway. We also determined that the extent of protein maturation through the secretory pathway correlated with a pathogenicity score obtained *via* Rhapsody, which we previously used to predict the extent of ERAD targeting of mutant forms of an unrelated ion channel (38, 39). Together, we suggest our pipeline provides a route to identify newly discovered disease-associated mutant forms of KCC2 as well as other channels and transporters with maturation defects.

## Results

### Residence of disease-associated mutations in KCC2 based on a homology model

To date, 12 disease-associated mutations in *SLC12A5* have been identified, and the molecular defects of A191V, L288H, S323P, M415V, R952H, and R1049C have been partially characterized (4, 7, 8, 9, 35, 36, 37). To visualize the distribution of the established disease-associated variants in the KCC2 primary sequence, we generated a linear model as well as a 2D model, showing the residence of each mutation with respect to the lipid bilayer (Fig. 1, *A* and *B*). Three mutations are predicted to alter residues within the transmembrane domains (A191V, L403P and M415V), four reside on an extracellular loop (L288H, W318S, S323P, and S376L) that is initially deposited into the ER lumen as KCC2 is synthesized, four reside on the intracellular C-terminal tail (R857L, R952H, R1048W, and R1049C), and one allele (G528D) is positioned between transmembrane domain (TMD) 8 and 9. The mutations shown in Figure 1*B* are also color-coded based on disease association, and although the majority of mutations are linked to epilepsy of infancy with migrating focal seizures, some mutations located on the C-terminal tail have been identified in ASD (R1048W) or in more than one disorder (R952H and R1049C).

Next, we mapped each mutation onto a 3D homology model of the predicted KCC2 structure (Fig. 1*C*; also see “Experimental procedures”). The homology model was obtained from the SWISS-MODEL database and was created using the KCC3 cryo-EM structure (25, 40), which allowed for visualization of the KCC2 dimer that most likely represents the active form of the transporters (2, 41, 42, 43, 44). Although none of the mutations appear to critically interfere with the dimer interface, many are buried within highly ordered regions of the protein, including the TMDs.

### Identification of maturation-defective disease-causing KCC2 mutants

As noted in the preceding sections, disease-associated mutations in KCC2 have been investigated to varying degrees, with some being examined in multiple studies (R952H and R1049C) (7, 8, 9), while others remain uncharacterized (W318S, S376L, R857L, and R1048W) (7, 36, 37). To better define the structure-function relationship between disease-causing mutants and their respective defects, we selected six mutants for analysis: A191V, L403P, M415V, R952H, R857L, and R1049C. The positions of the altered residues reside in both the TMD (thus potentially affecting ion transport), as well as in the intracellular domain, which is associated with the regulation of cell surface residence and cytoskeletal interactions (28, 31, 32, 45, 46). Some of the amino acid alterations might also hinder protein folding, an outcome that would retain the proteins in the ER and potentially target them for ERAD. The logic underlying the selection of these mutations is also detailed further below.

**Figure 2:**
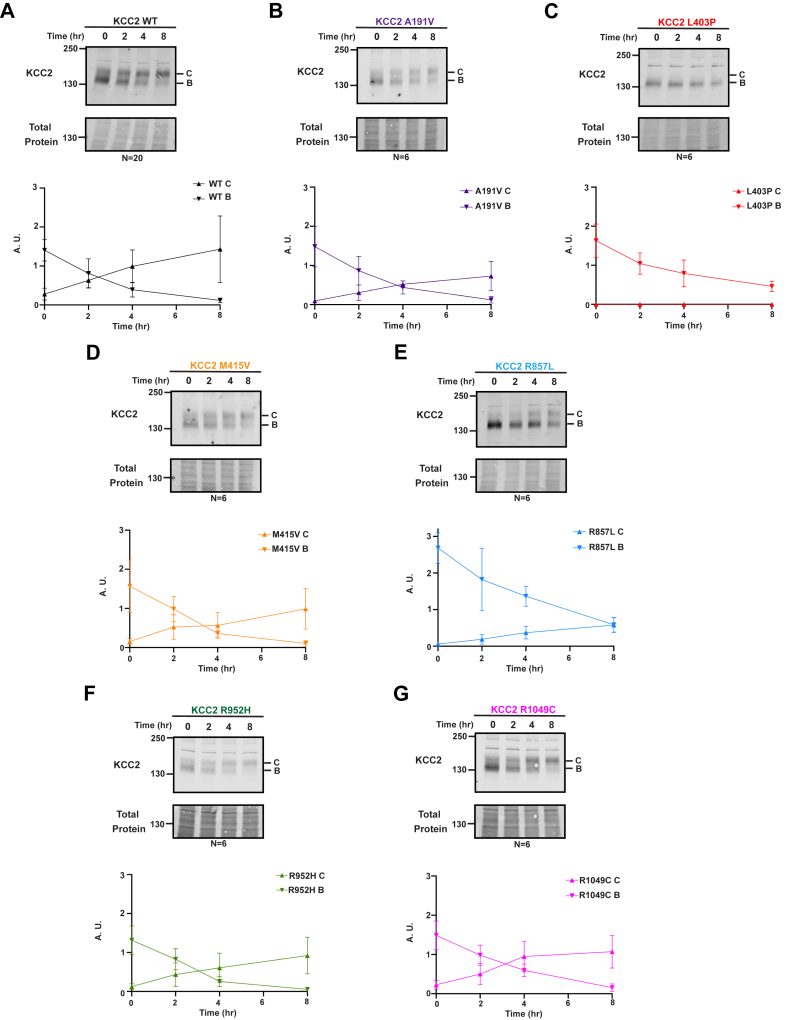
**The L403P and R857L KCC2 mutants mature inefficiently.** Cycloheximide chase analysis of transfected HEK293 cells expressing WT or mutant KCC2. Data for WT KCC2 (*A*) or the indicated mutant forms of KCC2 (*B*–*G*) are shown. Quantitative graph of KCC2 is shown below each respective blot. Immature KCC2 is designated B while Golgi-modified mature KCC2 protein is labeled *C*. Total protein is shown as the loading control. N values are indicated beneath each Western blot and represent independently transfected cell populations. Values are normalized to the total protein loading control. For all experiments, data represent the means ± SD.

Site-directed mutagenesis was applied to a plasmid designed for the transient expression of HA- and mCherry-tagged KCC2. This construct has been used in prior studies in mouse neuroblastoma (N2A) and HEK293 cells to assess KCC2 cell surface localization, activity, and maturation through the secretory pathway (8, 47). By assessing KCC2 biogenesis in HEK293 cells, we showed that WT monomeric KCC2 exists in both an immature core glycosylated state (denoted “B” on western blots) that contains N-linked glycans acquired in the ER, as well as a mature complex glycosylated form (denoted “C” on western blots) that contains elaborated glycans in the Golgi (Fig. 2*A*). To observe the maturation of KCC2 over time, we administered cycloheximide to inhibit protein synthesis and collected cellular lysates at 0, 2, 4, and 8 hours. As expected, WT KCC2 matured from its core glycosylated form to its complex glycosylated form. Ultimately, almost all the starting material was converted to the C form after 8 h, thereby demonstrating efficient maturation of nascent ER-synthesized KCC2 to the Golgi.

To determine whether the presence of each disease-associated mutation impacts KCC2 maturation, the results from cycloheximide chase assays were then compared between the six selected mutants (Fig. 2, *B*–*G*) and the WT protein. A range of results was observed, with some mutant proteins maturing similarly to WT KCC2 while others remained in the B form throughout the chase. For example, L403P (Fig. 2*C*) exhibited the greatest maturation defect, as the C form was absent even after 8 h. This suggests that the L403P mutation exerts a catastrophic impact on protein folding so it fails to acquire Golgi modifications. This result is consistent with prior studies that channel activity is also absent in HEK293 cells expressing the protein (35). Interestingly, other amino acid alterations (*e.g.*, A191V and R857L; Figure 2, *B* and *E*) had more modest effects on maturation since the transition from B to C was delayed and—in contrast to the WT protein—occurred closer to the 4- and 8-h timepoints, respectively (see quantitative graphs). It is also noteworthy that L403P resides in a TMD (Fig. 1*B*) and the more severe effect of this mutation may be due to the helix-breaking properties of proline, which when positioned within the TMD, is anticipated to strongly impact protein folding. In contrast, R857L is located in the intracellular C-terminal tail (Fig. 1*B*) and is predicted to form hydrogen or electrostatic bonds with a nearby serine (S825) and two aspartic acid residues (D827 and D1066). Therefore, it is perhaps unsurprising that substitution of the polar/charged R-group in arginine for a hydrophobic leucine side chain would disrupt the intramolecular electrostatic and polar interactions in this region. Together, these data suggest that the underlying cause of the diseases associated with the L403P, A191V, and R857L alleles is due, at least in part, to protein misfolding and impaired trafficking to the Golgi.

Interestingly, R952H and R1049C matured in a WT-like manner, despite being implicated in more than one disease (Fig. 2, *F* and *G*). When total KCC2 protein was considered in this experiment, R952H appeared to be expressed at somewhat lower steady-state levels, even though its stability over time was similar to each of the other mutant proteins as well as to the WT protein (compare 0 timepoint and time courses). As noted above, these alleles affect phosphorylation at S940, suggesting they alter the regulation of KCC2 but not its structure/biogenesis.

### KCC2 disease-associated mutants are variably but only modestly targeted for ERAD

After a plasma membrane—targeted protein is synthesized by ER-associated ribosomes and inserted into the ER, it begins to fold into its native structure and then most commonly progresses to the Golgi to acquire additional posttranslational modifications (48, 49, 50). Alternatively, if protein folding or assembly is compromised, the immature membrane protein can be targeted for ERAD. ERAD is a multistep process that involves the recognition of protein substrates by molecular chaperones, ubiquitination by E3 ligases, retrotranslocation into the cytosol by the p97 AAA—ATPase complex, and the ultimate breakdown of the ubiquitinated protein *via* the proteasome (51, 52, 53).

**Figure 3:**
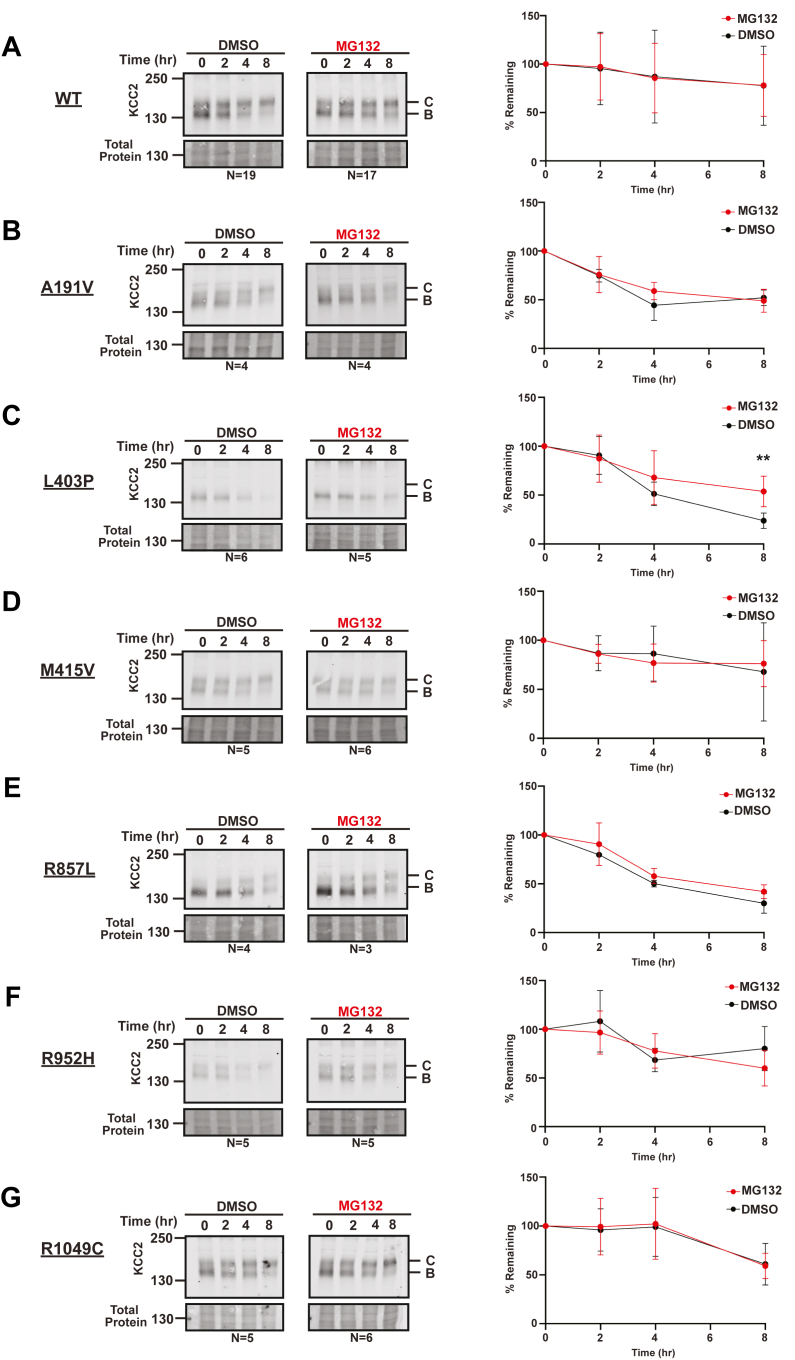
**KCC2 disease-associated mutants are not targeted for ERAD.** Cycloheximide chase analysis of transfected HEK293 cells expressing WT or the indicated KCC2 mutant. Data for WT KCC (*A*) or the indicated mutants (*B*–*G*) are shown. Cells were treated with MG132 or vehicle (DMSO). Quantitative immunoblot analysis of KCC2 protein levels at each timepoint are shown for WT (no significance), A191V (no significance), L403P (*p* = 0.0082), M415V (no significance), R857L (no significance), R952H (no significance), and R1049C (no significance). N values are indicated beneath each Western blot and represent independently transfected cell populations. Total protein was used as a loading control. Values are normalized to the total protein loading control. Data were analyzed using a Student's two-tailed *t* test and are shown as the means ± SD; ∗∗ = *p* < 0.01.

We recently reported that WT KCC2 is relatively stable when ectopically expressed in HEK293 cells, leading to only modest degradation *via* the ERAD pathway (47). Instead, KCC2 is turned over after retrieval from the cell surface (2). For some proteins (such as the sodium chloride cotransporter, NCC, another SLC12 family member), the presence of disease-associated mutations significantly destabilizes the protein, so it is delivered to the ERAD machinery (54, 55). To determine whether this was also the case for the KCC2 mutants examined in this study—a result that would suggest disease presentation is associated with ERAD, as seen for >70 other diseases (56)—we conducted cycloheximide chase assays in the presence of a proteasome inhibitor (MG132) or a vehicle control (DMSO) (Fig. 3). Interestingly, only L403P KCC2 was modestly stabilized when the proteasome was inhibited, and this effect was exclusively observed at the 8 h timepoint (Fig. 3*C*). Therefore, in contrast to the ER quality control event underlying the pathogenesis of several other ion channel– and transporter-associated diseases (56), ERAD does not appear to be the primary culprit underlying KCC2-linked disease presentation.

### Select disease-associated KCC2 variants are depleted from the cell surface

The data presented above establish that a subset of KCC2 mutations causes a maturation defect, and the protein acquires few if any Golgi-derived glycans. Yet, an analysis in rat hippocampal neurons found that some membrane proteins, including KCC2, are present in their core glycosylated state on the cell surface (57). This is indicative of a Golgi bypass mechanism in which a portion of the KCC2 protein is delivered to the plasma membrane without transiting through the entire Golgi apparatus. Notably, KCC2 is not the only member of the CCC family to exhibit this phenomenon: experiments in COS7 cells found that NKCC1 and NKCC2 also utilize this noncanonical pathway (58, 59).

**Figure 4:**
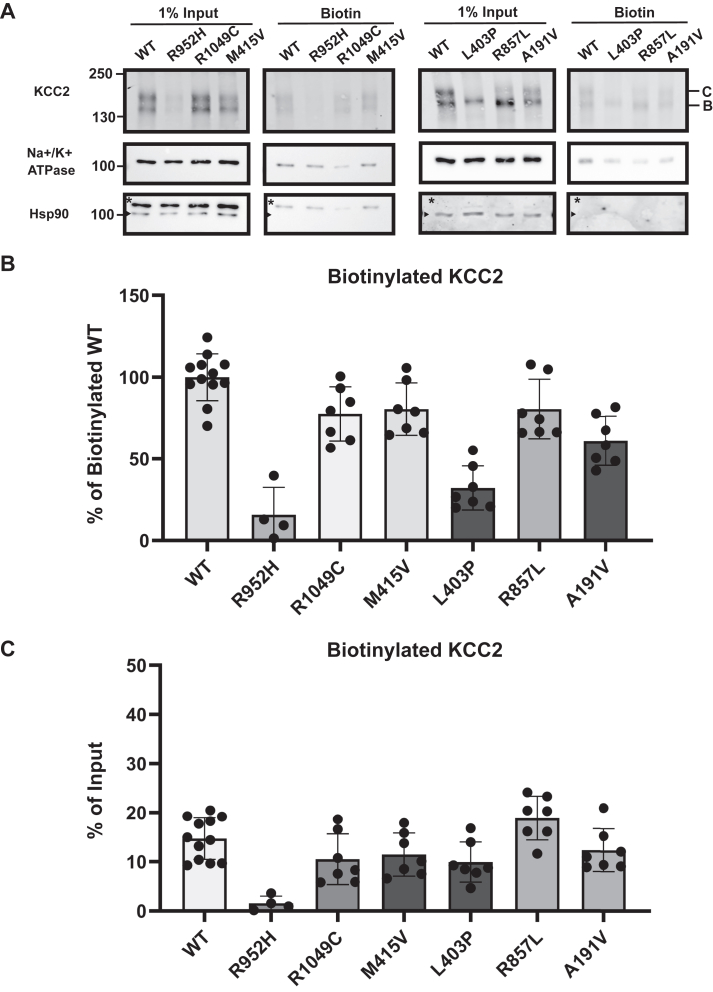
**Select KCC2 mutants are depleted from the cell surface**. *A*, Western blot analysis of cell surface biotinylation assays performed in HEK293 cells transiently expressing WT (N = 12), L403P (N = 7), R857L (N = 7), A191V (N = 7), R952H (N = 4), R1049C (N = 7), or M415V (N = 7) KCC2. The Na+/K + ATPase was detected as a plasma membrane protein control. Hsp90 was detected as a cytosolic protein control (see arrow, ∗ denotes variable levels of antibody stripping efficiency after detecting the Na+/K + ATPase). N values represent independently transfected cell populations. *B*, quantitative immunoblot analysis of the biotinylated R952H (*p* = 0.00028), R1049C (*p* = 0.012), M415V (*p* = 0.021), L403P (*p* < 0.0001), R857L (*p* = 0.036), and A191V (*p* = 0.00012) mutants relative to biotinylated WT KCC2. Values were normalized to 100, which represents the mean of the WT values. *C*, quantitative immunoblot analysis of the biotinylated R952H (*p* < 0.0001), R1049C (no significance), M415V (no significance), L403P (*p* = 0.029), R857L (no significance), and A191V (no significance) mutants normalized to their respective input values and compared to WT KCC2. Data are shown as the means ± SD. Data were analyzed using a Student's two-tailed *t* test.

To explore whether the apparent maturation defect we observed also compromises the ability of the protein to reach the cell surface, we conducted cell surface biotinylation assays in transfected HEK293 cells (Fig. 4). As controls, we also examined the susceptibility of an abundant cytosolic protein (Hsp90) and an established membrane protein (Na+/K+ ATPase) in the input and biotinylated/cell surface protein samples.

First, while Hsp90 was detected in the input lanes in Figure 4*A*, the protein was absent after precipitation with avidin beads. In contrast, the Na+/K+ ATPase was present in both fractions. Second, consistent with aforementioned published data (57), we observed both immature and mature glycosylated KCC2 at the cell surface (Fig. 4*A*). Specifically, both the B and C glycosylated forms of WT KCC2 were detected in the input and biotinylated (*i.e.*, cell surface) protein samples isolated after avidin-affinity pull-downs (see “Experimental procedures”). When we then examined the residence of disease-associated KCC2 mutants, the amount of total KCC2 at the cell surface was either near WT levels (R1049C, M415V, A191V, and R857L) (Fig. 4, *A* and *B*) or there was significantly less KCC2 protein detected at the plasma membrane (R952H and L403P). To account for any differences in protein expression, we then normalized the amount of biotinylated KCC2 at the cell surface to its respective input value (Fig. 4*C*). Statistical analyses of these data found that the R952H and L403P mutants were significantly depleted at the cell surface relative to WT KCC2. These results are consistent with prior studies in which cell surface levels of KCC2 variants were assessed (8, 9, 35, 36). In the case of R952H, reduced cell surface levels are consistent with the effect of the mutation on KCC2 phosphorylation and membrane stability (9). L403P, which appeared to be defective in maturation to the greatest degree (Fig. 2*C*), was also depleted from the cell surface. Of note, only the ER-modified form was present at the cell surface (Fig. 4*A*), suggesting that L403P evades the Golgi and traffics to the cell surface to some degree. Variable amounts of the B form corresponding to other mutants were similarly detected at the cell surface.

### Development of a stable and inducible KCC2 HEK293 expression system

To date, KCC2 disease-associated mutations have only been studied using transient transfection techniques in HEK293 cells, but the lack of controlled expression has the potential to “overload” the ER, induce a secondary stress response, and support illegitimate protein transport. To overcome these potential artefacts, we created stable HEK293 lines in which KCC2 is expressed under a doxycycline inducible promoter. To this end, clonal populations from single cells stably expressing KCC2 in an inducible fashion were isolated and selected as described in the Experimental Procedures. Based on key results presented above, we chose to create stable lines for WT KCC2 and the two mutants that lacked Golgi-associated glycans to the greatest degree, that is, L403P and R857L. These alleles include one that has been partially characterized (L403P) and one that has never been characterized (R857L). As anticipated, the inducible expression of WT, L403P, or R857L KCC2 in the stable cell lines was lower than that seen in the transiently transfected cells (Fig. S1). The stable cell lines also exhibited reduced levels of the ER molecular chaperone, BiP, an Hsp70 homolog whose expression is elevated by ER stress (60).

**Figure 5:**
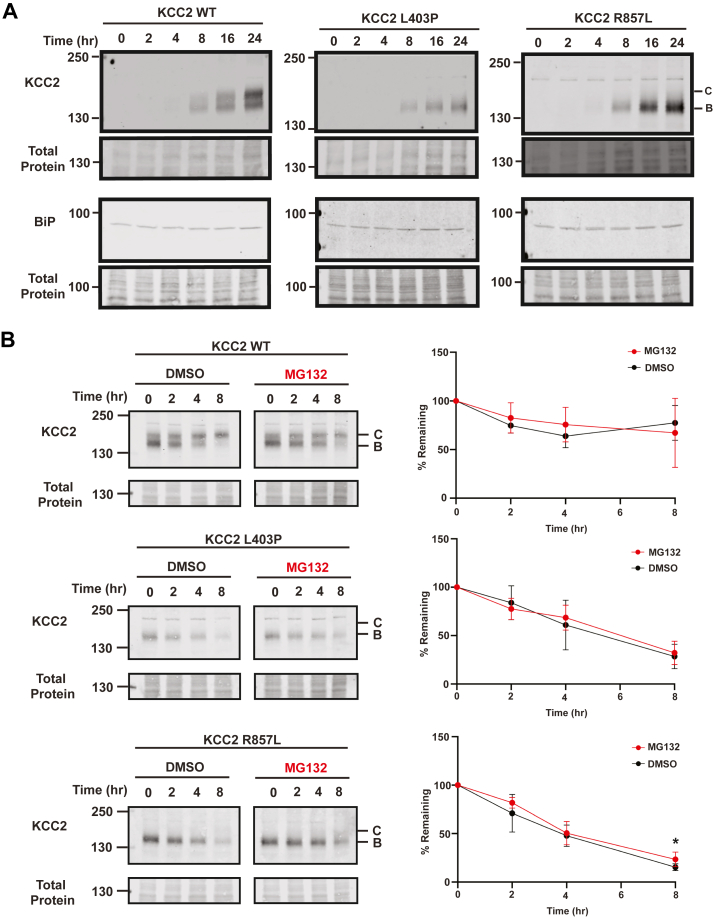
**KCC2 L403P and KCC2 R857L maturation is impaired in stable HEK293 cell lines.***A*, Western blot analysis of inducible KCC2 expression in stable HEK293 cells. Expression of KCC2 was induced with doxycycline for the indicated number of hours (hr). BiP was detected as an indicator of cell stress. Total protein was used as a loading control. *B*, cycloheximide chase analysis of HEK293 cells stably expressing WT, L403P, or R857L KCC2. Cells were treated with MG132 (*right*) or vehicle (DMSO) (*left*). Graph of quantitative immunoblot analysis of total KCC2 protein levels at each timepoint for WT (no significance), L403P (no significance), and R857L (*p* = 0.048) (*right*). N = 6 for all replicates. Total protein was used as a loading control, and values are normalized to the total protein loading control. Data were analyzed using a Student's two-tailed *t* test and are shown as the means ± SD; ∗ = *p* < 0.05.

We next treated each stable line with doxycycline and examined KCC2 expression over the course of 24 h (Fig. 5*A*). To ensure that a secondary stress response was absent, we again monitored the presence of BiP. In each stable cell line, KCC2 expression was detected by Western blot after 8 h of doxycycline treatment, and protein levels continued to rise for ∼24 h. The WT protein underwent Golgi-associated maturation to a significant degree since both the immature B and mature C glycosylation states were observed, especially at later timepoints (16 and 24 h). As seen in the transiently transfected cells, Golgi-associated glycans (*i.e.*, the C form) were again absent when the L403P protein was examined, even after 24 h. Interestingly, R857L maturation was highly compromised, even though limited band C/Golgi-modified species were present after transient transfection (Fig. 2 *E*). One explanation for the presence of band C R857L is that the ER was overloaded after transient transfection, allowing R857L to leak into the Golgi. Finally, it is noteworthy that in no case were elevated levels of BiP evident in the stable lines.

To confirm that WT KCC2 underwent ER- and Golgi-associated glycosylation, we treated cells with endoglycosidase H (endoH), an enzyme that cleaves immature ER-modified glycans (band B) but is unable to cleave more complex, mature Golgi-modified glycans (band C) (61). As shown in Fig. S2, endoH treatment of WT KCC2 led to a more rapidly migrating species that derived from band B KCC2. In contrast, the migration of band C was unaffected (Fig. S2). Like the WT protein, the migration of the L403P and R857L band B species was similarly enhanced, but band C species were again absent, consistent with the data shown in Figure 5*A*. In addition, the migration of the B band in WT, L403P, and R857L KCC2-expressing cells was identical after endoH treatment.

### Neither L403P nor R857L KCC2 are targeted for ERAD

We next assayed KCC2 maturation and stability *via* cycloheximide chase assays in the stable inducible cell lines. For these efforts, KCC2 expression was induced for 9 h prior to the addition of cycloheximide, and ERAD dependence was again assessed by treating cells with MG132 or DMSO. Consistent with data in transiently transfected cells (Fig. 3*A*), WT KCC2 was stable (Fig. 5*B*). Interestingly, the L403P mutant was again unstable but MG132 treatment had no effect on protein turnover. R857L was similarly unaffected by MG132 treatment, with the exception of modest stabilization at the 8 h timepoint (Fig. 5*B*). To test whether the proteins might instead be targeted for lysosome-associated degradation, we repeated the cycloheximide chase assays with these mutants, except bafilomycin A1, a drug that disrupts lysosome acidification and function (62), was added (Fig. S3). We found that bafilomycin increased the steady-state (*i.e.*, zero time point) levels of L403P and R857L KCC2 ∼3-fold, consistent with lysosome-dependent degradation. The lack of a significant effect on the apparent rate of turnover over the time course may reflect compensatory action of the proteasome or other degradative pathways during the cycloheximide chase, as observed in studies of other CCC transporters (see, *e.g.*, (54)). In fact, KCC2 is known to be acted upon by other proteases under some conditions (see Discussion).

### L403P and R857L are depleted from the cell surface in stable cell lines

**Figure 6:**
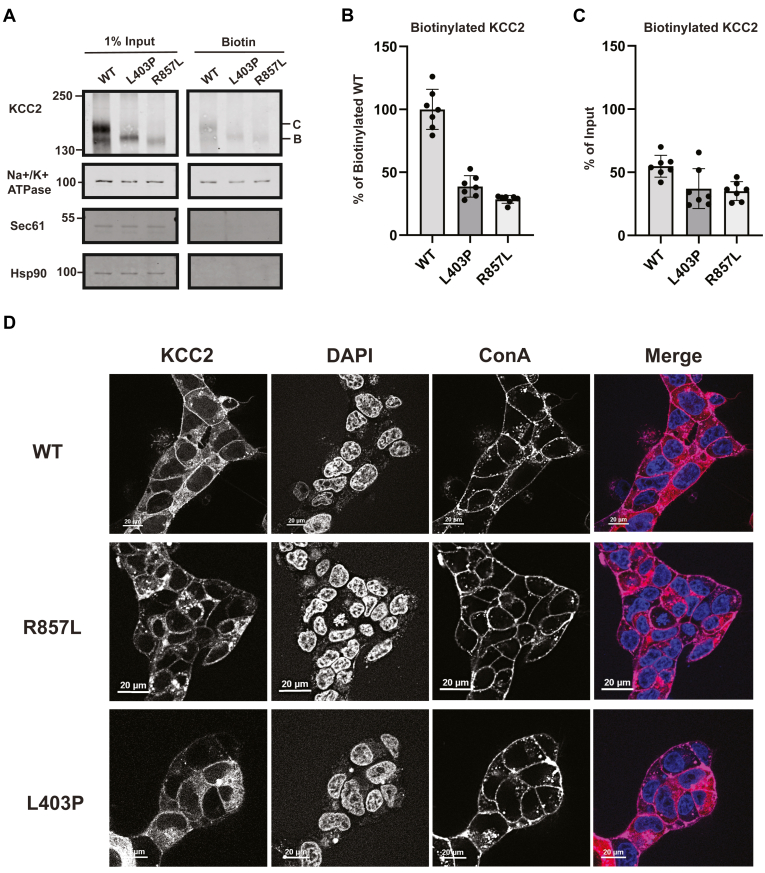
**The levels of the L403P and R857L KCC2 mutants are reduced at the cell surface in stable HEK293 cells.***A*, Western blot analysis of cell surface biotinylation assays performed in HEK293 cells stably expressing KCC2 WT, L403P, or R857L. The Na+/K + ATPase was detected as a membrane protein control. Hsp90 was detected as a cytosolic protein control. Sec61 was detected as an ER membrane protein control. N = 7 for all replicates. *B*, quantitative immunoblot analysis of biotinylated L403P (*p* < 0.0001) and R857L (*p* < 0.0001) mutants relative to WT KCC2. Mutant values were normalized to 100, which represents the mean of the WT values. *C*, quantitative immunoblot analysis of biotinylated L403P (*p* = 0.022) and R857L (*p* = 0.00064) mutants normalized to their respective input values and compared to WT KCC2. Data are shown as the means ± SD. Data were analyzed using a Student's two-tailed *t* test. *D*, live cell imaging of HEK293 cells stably expressing WT, L403P, or R857L KCC2 with an mCherry tag (*red*). The nucleus is labeled with DAPI (*blue*), and the plasma membrane is labeled with Concanavalin A (ConA, *magenta*). Scale bars are shown in the *bottom left corner* of each panel.

One anomaly from the transient transfection studies was that the levels of cell surface biotinylated WT and R857L KCC2 were similar (Fig. 4*C*), even though the acquisition of band C R857L was significantly delayed (Fig. 2*E*). To examine whether this phenomenon was evident after controlled expression, we performed cell surface biotinylation assays with the inducible KCC2 stable cell lines. Hsp90 and the Na+/K+ ATPase again served as cytosolic and plasma membrane protein controls, respectively (Fig. 6*A*). To provide an additional control, we also assessed Sec61α, an ER-resident membrane protein that serves as a key component of the ER translocon and is absent from the plasma membrane (63). As expected, Sec61α was detected in the input samples but not in the cell surface/biotinylated samples. After doxycycline induction of WT, L403P, and R857L KCC2 for 16 h to maximize expression, we again detected biotinylated band B and band C WT KCC2 at the cell surface (Fig. 6*A*), consistent with the data in Figure 4*C* and with previous hints that KCC2 may utilize a Golgi bypass mechanism (57). In addition, consistent with the results in Figure 5*A*, the immature forms of L403P and R857L were also detected on the cell surface but to a more limited extent when compared to surface-resident/biotinylated WT KCC2 (Fig. 6*B*). To account for differences in protein expression, biotinylated KCC2 levels were also normalized to their input values prior to analysis, and both mutants were still depleted from the cell surface relative to WT KCC2 (Fig. 6*C*). Intriguingly, closer inspection of biotinylated WT KCC2 reveals a consistent ratio of B and C compared to the input, suggesting that both pathways—the canonical secretory pathway and the Golgi bypass pathway—are equally efficient in transporting KCC2 to the plasma membrane. This aligns with prior work examining other substrates: bypassing the Golgi is just as effective, if not more so, at transporting cargo to the cell surface (64, 65). In addition, the discrepancy between results in the transiently transfected *versus* the stable lines for the R857L mutant can once again be explained by the more moderate protein levels in the stable cells and the absence of ER stress (Fig. S1).

To support data on the observed differences in cell surface residence between WT KCC2 and the L403P and R857L variants, we took advantage of the mCherry tag appended to the N-terminus of KCC2 and conducted live cell imaging experiments (Fig. 6*D*). WT KCC2 was clearly detected at the plasma membrane—as shown *via* colocalization with a fluorescent concanavalin A conjugate (see Experimental procedures)—and some protein was also more diffusely distributed inside of the cell. In contrast, R857L and L403P residence at the plasma membrane was reduced and an elevated level of protein resided in the cell interior. Interestingly, the R857L KCC2 mutant was found in intracellular puncta, which may reflect inclusions in the ER or in other components of the secretory pathway. Regardless, the imaging data align with the cell surface biotinylation data and are consistent with decreased acquisition of Golgi modifications by these KCC2 mutants.

### The maturation of KCC2 mutant alleles can be computationally predicted

We previously showed that computational tools can predict the pathogenicity of disease-associated mutations in an ion channel (38, 39), more specifically, the renal outer medullary potassium (ROMK) channel, which is one of several proteins that regulates potassium homeostasis in the kidney (66, 67). Mutations in ROMK have been identified in individuals with Bartter syndrome type II, a severe salt-wasting disorder (68, 69). To perform this analysis, we utilized Rhapsody, a validated machine-learning pathogenicity predictor that uses sequence conservation and structural accessibility, along with an elastic modeling network that incorporates dynamics-based features to analyze the potential pathogenicity of every possible amino acid and at every position in a protein sequence (70) (also see Experimental procedures). Rhapsody assigns a score from 0 to 1 for each substitution, with higher values representing a potentially more pathogenic mutation. When ROMK was analyzed, Rhapsody values approaching 1 correlated with mutations exhibiting reduced stability and enhanced ERAD in HEK293 cells (39). Therefore, we next asked if Rhapsody might alternatively be able to predict KCC2 maturation defects. Because the results from the transient transfection analyses of the WT protein and the six KCC2 mutants provided a richer dataset—and because the levels of band C generally correlated with protein maturation efficiency in the ER—the results in Figure 2 were used for this analysis (but see below).

**Figure 7:**
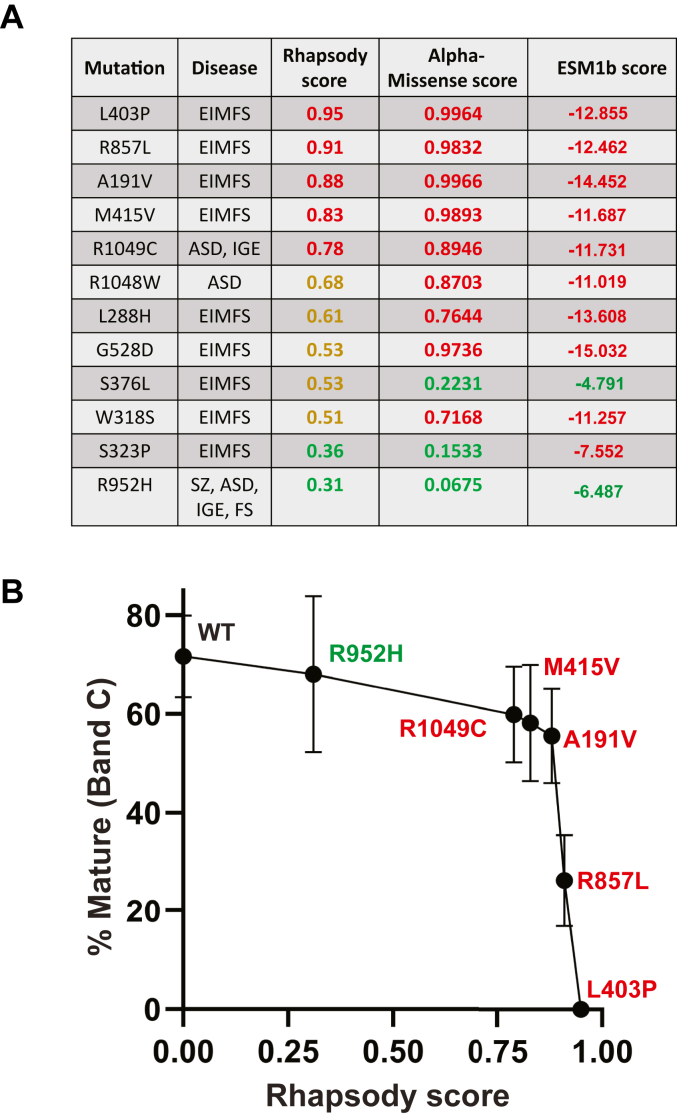
**The Rhapsody score correlates with KCC2 ER to Golgi maturation.***A*, pathogenicity predictions for each disease-associated KCC2 mutant. Disease abbreviations are as follows: epilepsy of infancy with migrating focal seizures (EIMFS), febrile seizures (FS), schizophrenia (Sz), autism spectrum disorder (ASD), and idiopathic generalized epilepsy (IGE). Predictions were made using Rhapsody, AlphaMissense, and ESM1b. Predicted values are colored coded according to their Rhapsody scores (*red* = highly deleterious, *yellow* = moderately deleterious, and *green* = benign), AlphaMissense scores (*red* = pathogenic and *green* = nonpathogenic), and ESM1b scores (*red* = pathogenic and *green* = nonpathogenic). *B*, quantitative analysis showing the maturation of monomeric KCC2 relative to the Rhapsody score for each mutation. % Maturation to the C band (*i.e.*, acquisition of Golgi-associated glycans) was measured for WT and each mutant KCC at the 4-h timepoint in cycloheximide chase assays shown in Figure 2. Mutants are color coordinated according to the severity of their Rhapsody scores shown in (*A*). Data are the means ± SD.

Cryo-EM structures of KCC2 have been reported (24, 25, 71), but because they lack the appropriate resolution to map disease-associated residues, we utilized a homology model based on the structure of the closely related KCC3 protein (see Experimental procedures) and then calculated Rhapsody scores for each of the 12 disease-associated KCC2 mutations (Fig. 7*A*). Based on prior work, Rhapsody scores >0.75 are predicted to be highly deleterious, scores between 0.75 and 0.5 are predicted to be moderately deleterious, and scores <0.5 are predicted to be benign (70).

Among the 12 variants, five are categorized as highly deleterious (L403P, R857L, A191V, M415V, and R1049C; Fig. 7*A*, values in red), five are predicted to be moderately deleterious (R1048W, L288H, G528D, S376L, and W318S; values in yellow), and two are thought to be benign (S323P and R952H; values in green). We then compared the extent of protein maturation among the mutants at the 4 h timepoint—which best captures the extent to which a protein is Golgi modified (Fig. 2)—with the Rhapsody score of each mutant. As shown in Figure 7*B*, an intriguing correlation was observed. In short, there appears to be a Rhapsody cut-off score, above which the maturation of ER-modified KCC2 to the Golgi is inhibited (Fig. 7*B*). More specifically, the rank order by Rhapsody for the L403P, R857L, and A191V alleles correlates directly with protein maturation.

A significant number of other predictive algorithms that measure the effects of missense mutations exist. While a full analysis of the >50 such programs (72) is beyond the scope of this study, we compared the Rhapsody data to the results obtained with two recent but widely used high-performance pathogenicity predictors, AlphaMissense and ESM1b (73, 74). In brief, AlphaMissense surveys population frequency, data from molecular modeling, amino acid–specific features, and structural information to assign a pathogenicity score, with a higher value denoting a more deleterious substitution. ESM1b uses sequence similarity and evolutionary relationships—and more specifically, potential ligand-binding sites, posttranslational modifications, intramolecular interactions, and structural cues—to assign a pathogenicity score. For ESM1b, a more negative value is deleterious. Based on the data shown in Figure 7, some consistencies between Rhapsody, AlphaMissense, and ESM1b are apparent, yet the relative ranking of the mutants is different. For example, both AlphaMissense and ESM1b score A191V, which exhibits a subtle defect in maturation (Fig. 2), as the most deleterious allele. This is surprising given the presumed modest effect of an alanine to valine substitution and conflicts with our data. However, Rhapsody correctly places this allele as exhibiting an intermediate effect between M415V and R857L. In sum, we suggest that Rhapsody can be used to predict the maturation efficiency of additional KCC2 variants as they are isolated and can perhaps be applied to proteins within the same ion transporter family.

## Discussion

In this study, we examined six disease-associated KCC2 mutations that are either uncharacterized or were partially characterized. The alleles were initially selected based on their residence within the protein and/or the range of diseases with which they are associated. We first measured WT and mutant protein maturation over time. The read-out for this ER quality control step was the acquisition of Golgi-associated glycans, based on a prior report (47). We found that maturation of some variants was attenuated (*e.g.*, A191V and R857L), and one mutant was unable to acquire these glycans (L403P). There was also general correlation between Golgi maturation and cell surface residence, as assessed by cell surface biotinylation in stable HEK293 cells. Overall, select KCC2 variants evaded Golgi modification and were inefficiently transported to the plasma membrane, as seen for numerous other cell surface proteins that pass through the secretory pathway.

A more unexpected outcome of our efforts was that the KCC2 mutants examined in this study were ERAD-resistant. The lone exception was data from a single timepoint when the stability of the L403P mutant was examined after transient transfection. Interestingly, L403P KCC2 exhibited the most significant maturation defect. Based on this result, we suggest that structural defects associated with the L403P mutant prevent its maturation to the Golgi but are insufficient to deliver the misfolded protein for ERAD. However, after lysosome function was attenuated with bafilomycin A1, significant L403P (and R857L) protein stabilization under steady-state conditions was evident (Fig. S3). These data align with previous reports that KCC2 is recycled from the plasma membrane and that, over time, a portion is turned over by lysosomal proteases (31, 46, 75). Yet, the time scale for endocytosis-associated degradation may be longer than that which was used in the cycloheximide chase, so the rate of KCC2 degradation by the lysosome after bafilomycin addition was negligible in this experiment. Notably, however, KCC2 degradation can be also preceded by cleavage at the C-terminus by calpain, which regulates cell surface expression and leads to lysosome delivery (43, 75, 76). Therefore, future efforts will seek to define the relationship between calpain-dependent quality control and other degradative pathways that mediate post-ER degradation of KCC2. More generally, the apparent resistance of KCC2 to ERAD may reflect the fact that KCC2 simply lacks a lumenal degron (77) that facilitates ERAD targeting.

To date, only a dozen disease-associated mutations in the gene-encoding KCC2 have been identified. Although this likely reflects the difficulty in isolating unique alleles associated with complex diseases—especially those in which genetics and environmental factors play contributing roles—the relatively small number of known disease-associated alleles in the KCC2-encoding gene suggests that many more alleles remain to be discovered. Because we found a direct correlation between the Rhapsody score and the amount of KCC2 that trafficked to the Golgi, we suggest that Rhapsody can be used to predict disease severity of uncharacterized KCC2 variants. This is an important endeavor given the plethora of mutations that continue to be identified in individuals with neurodevelopmental disease (2, 4, 78). This goal is also vital given the expanding number of chemical chaperones that facilitate ER protein folding, exit from the ER, and protein function (79, 80).

In addition to Rhapsody, great effort has been made toward the development of other computational tools that predict the severity of single amino acid substitutions in proteins implicated in human disease. This has led to the creation of multiple pathogenicity predictors, including AlphaMissense and ESM1b (73, 74). We chose to include Rhapsody in our analysis since we previously used this platform to analyze mutations in ROMK, an ion channel associated with Bartter syndrome. The outcome of our studies was that Rhapsody scores accurately identified mutations routed to the ERAD pathway (39). Another value of Rhapsody is that single amino acid variants, which may or may not be associated with disease, can be rapidly computationally screened. This may, in principle, aid physicians and genetic counselors to predict disease outcome. It is also noteworthy that Rhapsody helped focus our attention on an unexplored disease variant (R857L, score = 0.91) that may have otherwise been overlooked. Indeed, R857L showed impaired maturation and reduced cell surface expression. In contrast, none of the variants located on the KCC2 extracellular loop (L288H, W318S, S323P, and S376L) had a high Rhapsody score and were thus considered moderately deleterious or benign (Fig. 7*A*). This observation suggests that perturbations in the extracellular loop may lack catastrophic effects on protein folding.

The molecular machinery that prevents KCC2 maturation to the Golgi and releases substrates from the ER has yet to be determined. Candidates for this pathway include chaperones such as the cytoplasmic Hsp70 and BiP, which play critical roles during ER quality control. Consistent with this hypothesis, we previously showed that Hsp70 is required for the ERAD of the sodium chloride cotransporter, NCC, which is a distant KCC2 relative (54, 55). The importance of this chaperone was underscored by the observation that the interaction of ER quality control factors with mutant forms of NCC were elevated compared to the WT protein. Other candidates include the ER chaperone-like lectins, such as calnexin and OS9, especially since the lectins play key roles in ER protein folding (81), and calnexin interacts with KCC2 after affinity purification in neurons (82). Moreover, OS9 and Hsp70 modulate the proteostasis of NKCC2 (83, 84), a related ion transporter family member, in mammalian cells. A thorough characterization of the KCC2 quality control machinery thus represents another future goal.

We provide evidence that KCC2 may utilize a Golgi bypass mechanism since both mature and immature protein were detected on the cell surface in HEK293 cells (Figs. 4 and 6). Another ion transducer, CFTR, was also shown to utilize this system, but the use of the Golgi bypass pathway was cell type–specific; namely, CFTR utilized a Golgi by-pass pathway in HeLa and HEK293 cells but not in BHK or CHO cells (85). Since immature core glycosylated KCC2 was detected on the cell surface in rat hippocampal cells (57) as well as on the surface of HEK293 cells, as seen in this study, it is unlikely that cell type–specific effects exist that differentially route KCC2 to the cell surface. The mechanisms that underlie this pathway are still being deciphered (86), but in time, it will be important to understand why KCC2 utilizes one pathway *versus* another.

The maturation defects we observed for KCC2 variants are evocative of observations seen in other disease-relevant proteins, such as CFTR. As noted above, the most common CFTR mutation that causes cystic fibrosis (F508del) prevents maturation from an immature core glycosylated state to the complex form over time (87), but in contrast to KCC2, F508del CFTR is delivered to the ERAD pathway. Endeavors to correct this defect were successful and led to the development of Trikafta, an FDA-approved treatment for cystic fibrosis (88). Trikafta is a combination therapy that includes compounds designed to correct protein folding/maturation and potentiate channel activity at the cell surface. Whether the maturation of KCC2 variants can similarly be corrected using chemical chaperones remains to be determined.

Finally, we emphasize that Rhapsody relies upon an available protein structure or homology model to render pathogenicity predictions (70). With continued improvements in experimental and computational protein structure predictions, it will be exciting to expand the use of Rhapsody for deleterious mutations across a wider variety of protein substrates. In tandem, the emergence of large human genome databases, such as ClinVar (89), UK Biobank (90), and TOPmed (91), have uncovered hundreds of thousands of disease-associated mutations within the human genome that have yet to be characterized. As these databases expand further, we anticipate that tools, such as Rhapsody, will become more critical with time. In fact, while this manuscript was in preparation, a previously unknown KCC2 variant (R231H) was identified in a patient with infancy with migrating focal seizure, and the protein exhibited reduced activity and cell surface expression in N2a cells (78). Interestingly, the Rhapsody score for this allele was 0.84, that is, it is predicted to be highly deleterious. Therefore, future endeavors to characterize this mutant would be worthwhile and may reveal a maturation defect similar to the high scoring mutants included in this study.

## Experimental procedures

### Site-directed mutagenesis

To create the six point mutations in the coding region of rat KCC2b (A191V, L403P, M415V, R857L, R952H, and R1049C), the QuickChange Site Directed Mutagenesis kit (Stratagene) was used per the manufacturer’s instructions. All oligonucleotide primers used are listed in Table S1. An HA- and mCherry-tagged rat KCC2b in a pcDNA3.1(+) plasmid (8) was used as a template for PCR mutagenesis, and site-specific mutations in the KCC2 sequence were confirmed by DNA Sanger sequencing (Plasmidsaurus).

### Human cell culture

HEK293H cells were cultured in Dulbecco's modified Eagle’s medium (DMEM) (Sigma-Aldrich) with 10% FBS at 37 °C in a 5% CO_2_ humidified incubator and were tested for *mycoplasma* contamination upon receipt. For transient transfection studies, a total of 0.6 × 10^6^ cells were plated into 6-well poly-L-lysine–coated plates. Once cells were at 70 to 90% confluency, transfection was performed using 4 μg of the KCC2-HA-mCherry plasmid indicated above. In brief, DNA and Lipofectamine 2000 (Invitrogen) were incubated in Gibco Opti-MEM media separately for 5 min at room temperature before being combined and incubated at room temperature for 20 min. After incubation, the DNA solution was added to the cells and plates were returned to 37 °C. Fresh media was added after 4 to 5 h. Experiments were conducted 24 h after transfection. The HEK293H cell line used in this study was last authenticated by the University of Arizona Genetics Core in December, 2016, using fragment analysis. This cell line was cloned from the original HEK293 cell line per the manufacturer (Thermo Fisher Scientific) after transformation with sheared human adenovirus type 5 DNA and exhibits superior adherent properties.

To construct HEK293H cells that stably express KCC2-HA-mCherry in a doxycycline-dependent manner, the T-Rex inducible protein expression system was implemented (Thermo Fisher Scientific). Per the manufacturer’s protocol, HEK293H cells were first transfected with the pcDNA6/TR plasmid and then blasticidin-resistant clones were isolated (92). Next, cells with pcDNA6/TR successfully integrated were transfected with a doxycycline-inducible expression plasmid containing KCC2-HA-mCherry. To construct this plasmid, which expressed WT KCC2 or contained the L403P or R857L mutations, the DNA inserts were cut with restriction enzymes AflII and EcoRI and subcloned into the doxycycline inducible vector provided by the manufacturer. Sequences were confirmed with DNA Sanger sequencing (Plasmidsaurus). Next, transfected cells were treated with zeocin and individual clones were isolated from 96-well plates *via* dilution. After 14 days of zeocin treatment, 24 individual clones were transferred into 12-well plates for expression analysis. To test for KCC2 expression, cells with robust growth (70–75% confluency, ∼12 clones for each construct) were treated with doxycycline (1 μg/ml) for 24 h prior to lysis and Western blot analysis, as described below. Cell lines that expressed KCC2 and showed minimal KCC2 expression without added doxycycline were retained (∼3 clones per construct). Finally, doxycycline induction time courses were conducted for each cell line and lysates were collected at 0, 2, 4, 8, 16, and 24 h post-induction. Lysates were subjected to Western blot analysis as described below, and BiP levels were assessed to monitor cell stress. Cell lines that did not trigger cell stress upon KCC2 induction were stored at −80 °C and aliquots were thawed and used for the experimental analyses reported below (1 clone per construct).

### Protein stability measurements

Cycloheximide chase assays were used to measure protein stability in transfected HEK293 cells and were conducted as previously described (54). In brief, to halt protein synthesis, cycloheximide was added at a final concentration of 50 μg/ml. At each timepoint, the media was aspirated, and cells were lysed with ice-cold TNT buffer (5 mM Tris, pH 7.2, 15 mM NaCl, 1% Triton X-100) with a complete protease inhibitor cocktail (Roche). The lysate was centrifuged at 13,000 RPM for 10 min at 4 °C and diluted in SDS sample buffer (6 mM Tris, pH 6.8, 1% β-mercaptoethanol, 2% SDS, 0.05 mg/ml bromophenol blue), and SDS-PAGE and Western blot analysis were performed as described below. For experiments in which the proteasome was inhibited, cells were pretreated with MG132 (25 μM) or DMSO (vehicle) for 1 h prior to the addition of cycloheximide. For experiments in which the lysosome was inhibited, cells were pretreated with bafilomycin A1 (100 nM) or DMSO (vehicle) for 18 h to achieve sufficient inhibition prior to the addition of cycloheximide. To ensure that protein synthesis remained inhibited for the duration of the assay, the same concentration of cycloheximide was added to each well and at each timepoint. Experiments conducted in stable cell lines were performed in the same manner, but the assay was performed 9 h after doxycycline (1 μg/ml) addition. Samples were again subjected to SDS-PAGE and Western blot analysis, as detailed below.

### Measurements of protein glycosylation

To assess protein glycosylation, the indicated stable KCC2-expressing HEK293 cell lines were grown, induced with doxycycline, and lysed as stated above. Lysates were treated with endoglycosidase H (endoH; Sigma-Aldrich) or an equivalent volume of water in 0.1 M sodium citrate, pH 5, as per the manufacturer’s recommendation. For Western blot analysis, samples were incubated at 37 °C for 2 h and resolved on 5% polyacrylamide gels for immunoblot analyses (see below).

### Cell surface biotinylation assays

HEK293H cells were seeded into 6-well poly-lysine–coated plates and transfected as described above. At 24 h post-transfection, cells were treated with 125 μg/ml cycloheximide for 2 h at 37 °C. The plates were then washed with cold 1× DPBS (Thermo Fisher Scientific) and incubated in 0.3 mg/ml EZ-Link Sulfo-NHS Biotin (Thermo Fisher Scientific) for 1 h on ice. After removal of media containing biotin, the HEK293H cells were detached with trypsin (TrypLE, Thermo Fisher Scientific), and free biotin was quenched with 100 mM glycine. Next, the cells were lysed in HEENG lysis buffer (20 mM Hepes pH 7.6, 1 mM EDTA, 1 mM EGTA, 25 mM NaCl, 1% Triton-X, 10% glycerol, and a protease inhibitor cocktail tablet) followed by centrifugation at 13,000 RPM in a microfuge at 4 °C and collection of the supernatant. Protein concentration was determined using the Pierce BCA protein assay kit (Thermo Fisher Scientific) with a bovine serum album protein standard, and each sample was normalized to contain 600 μg of protein. For the input sample, 1% of the lysate was collected prior to incubating the lysates in 35 μl Piece NeutrAvidin-agarose beads (Thermo Fisher Scientific) overnight at 4 °C. After washing the beads with cold 1× DPBS, the beads were resuspended in SDS sample buffer and analyzed by SDS-PAGE and Western blot as described below. Experiments conducted in stable cell lines were performed as described above 16 h after doxycycline (1 μg/ml) induction.

### SDS-PAGE and western blot analysis

For SDS-PAGE and Western blot analysis, all cellular lysates in sample buffer were incubated at 37 °C for 10 min prior to being loaded onto 5% or 10% SDS-PAGE gels. KCC2 was detected using a rabbit anti-KCC2 polyclonal primary antibody (1:1000, Sigma-Aldrich) and a goat anti-rabbit IRDye-labeled secondary antibody (1:5000, LI-COR). Hsp90 was detected with a mouse anti-Hsp90 monoclonal primary antibody (1:1000, Enzo Life Sciences), and the Na^+^/K^+^ ATPase was detected with a mouse anti-Na^+^/K^+^ ATPase monoclonal primary antibody (1:1000, Developmental Studies Hybridoma Bank). Both primary antibodies were then decorated using a goat anti-mouse IRDye-labeled secondary antibody (1:3000, LI-COR). BiP was detected with a rabbit anti-BiP monoclonal primary antibody (1:1000, Cell Signaling Technologies) and Sec61α was detected with a rabbit anti-Sec61α polyclonal primary antibody (at 1:1000; Invitrogen). Detection of both BiP and Sec61α required the goat anti-rabbit secondary antibody described above. Finally, protein signals were uncovered on a BioRAD Universal Hood II Imager or a LI-COR Odyssey CLX imaging system. For all analyses, a Pageruler Plus Prestained Protein ladder (10 kDa to 250 kDa) was used to assess protein molecular weight, and a REVERT Total Protein Stain (LI-COR) was used as a loading control. The specificity of each antibody was validated against the predicted molecular weights of each protein and images provided by the manufacturer.

### Live cell imaging

HEK293 cells stably expressing WT or the indicated KCC2 mutant were seeded in 35 mm glass bottom Mat Tek dishes. At 16 h post-induction with doxycycline (1 μg/ml), cells were incubated at 37 °C in 5% CO_2_ for 20 min with DAPI (15 μg/ml, Thermo Fisher Scientific) and for 5 min with Alexa Fluor 647 conjugated Concanavalin A dye (Thermo Fisher Scientific). After incubation, cells were washed with fresh media and incubated in DMEM containing 10% FBS and under the same environmental conditions. The DMEM lacked phenol red (Sigma-Aldrich) to prevent background fluorescence. The cells were then immediately imaged using a 60× oil immersion lens on a Nikon A1R confocal microscope equipped with a temperature and CO_2_ controlled live cell incubator. Images were captured and analyzed with the NIS Elements software. For each image, the specificity of each antibody was validated against images provided by the manufacturer.

### Homology models and computational analyses to predict mutation pathogenicity

The KCC2 homology model based on the available human sequence (Uniprot #Q9H2X9) utilized in this study was obtained from the SWISS-MODEL database (40) and was created using KCC3 (PDB: 6Y5R) as a template (25). The KCC2 and KCC3 amino acid sequences are 73% identical and the KCC3 template (QSQE score = 0.94) was obtained by cryo-EM, as reported (25). The model covers 95% of the sequence (amino acids 88–1133) and has a QMEANDisCo Global score of 0.70 ± 0.05 and a GMQE score of 0.65. No ligands were included in the creation of this model. The structure was visualized and rendered using PyMOL (version 2.2.3).

Rhapsody is a computational machine-learning tool that predicts the pathogenicity of each amino acid substitution within a protein sequence. It incorporates sequence-, structure-, and dynamics-based information from a submitted protein structure to score potential pathogenesis. The Rhapsody algorithm, development, and analysis of ion channels were previously described (38, 39, 70). To predict the severity of disease-associated mutations previously identified in KCC2 (4), the KCC2 homology model (see above) was submitted to the Rhapsody server (rhapsody.csb.pitt.edu). The output provided a Rhapsody score for each amino acid substitution on a scale from 0 (no predicted effect on protein pathogenicity) to 1.0 (the most severe effect on protein pathogenicity). For comparison, we also used AlphaMissense, another pathogenicity predictor that incorporates population frequency data, molecular modeling based on protein sequence, and structural information from an AlphaFold-derived system to assess amino acid substitutions (73, 93). Scores are similarly given on a 0 to 1 scale, with values closest to 1 having the highest predicted pathogenicity. Finally, we utilized ESM1b, a computational protein language model that does not utilize a homology model for its pathogenicity predictions, but instead primarily uses evolutionary factors (74, 94). ESM1b also takes into consideration potential ligand-binding sites, posttranslational modifications, intramolecular interactions, and structural cues. Predictions for KCC2 were obtained by submitting the protein sequence (Uniprot #Q9H2X9) to the ESM1b interface at https://huggingface.co/spaces/ntranoslab/esm_variants. Scores with values less than −7.5 are considered pathogenic while scores over −7.5 are predicted to be nonpathogenic.

### Statistical analysis

For Western blot quantification, densitometry was carried out using the NIH ImageJ software (95), and raw integrated densities were analyzed in Microsoft Excel. GraphPad Prism software version 8.2.1 was used to create all graphs. Statistical comparisons between experimental and control protein levels at each cycloheximide chase timepoint were determined by a Student’s two-tailed *t* test. For cell surface biotinylation experiments that compared biotinylated WT *versus* mutant KCC2, the levels of the biotinylated protein were normalized to 100, which represents the mean of WT biotinylated protein levels and were also analyzed using a Student's two-tailed *t* test for each replicate. To account for differences in KCC2 expression in the cell surface biotinylation experiments, WT and mutant values were then normalized to their respective input levels (% input) before analysis with a Student’s two-tailed *t* test. No blinding was performed for any of the experiments, and no test for outliers was conducted. For all experiments, at least three biological replicates were conducted, and N values are indicated within each figure or figure legend. Each biological replicate represents a population of independently transfected or independently drug-treated HEK293 cells. In all figures, data represent the means ± SD and ∗ = *p* ≤ 0.05, ∗∗ = *p* ≤ 0.01, ∗∗∗ = *p* ≤ 0.001.

## Data availability

All data are contained within the manuscript.

## Supporting information

This article contains supporting information.

## Conflict of interest

The authors declare that they have no conflicts of interest with the contents of this article.
