# Supplementary material for: Inefficient maturation of disease-linked mutant forms of the KCC2 potassium-chloride cotransporter correlates with predicted pathogenicity
Source: J Biol Chem. 2025 Mar 10;301(4):108399. doi: 10.1016/j.jbc.2025.108399 (PMC12001125; doi:10.1016/j.jbc.2025.108399)
Supplement: Supporting information [file mmc1.pdf]

Mutation	Forward Primer Sequence	Reverse Primer Sequence
A191V	5’ CCTGGGCACTACCTTTGTTGGGGCTATTAC 3’	5’ GTACATAGCCCCAACAAAGGTAGTGCCCAGG 3’
L403P	5’ CTCCTACTTCACCCTGCCTGTTGGCATCTATTTCC 3’	5’ GGAAATAGATGOCAAGAGGCAGGGIGAAGTAGGAG 3’
M415V	5’ CAGTCACAGGGATCGTGGCTGGCTCGAAC 3’	5’ GTTCGAGCCAGCCACGATCCCTGTGACTG 3’
R857L	5’ GAGGAAATGCAAAATCTGATCTTCACCGTGGCGC 3’	5’ GCGCCACGGGAAGATCAGCATTTTGCATTTCCTC 3’
R952H	5’ CAACACTCGGCTCCACCTCAATGTTCCCG 3’	5’ CGGGAACATTGAGGTGAGCCGAGTGTTG 3’
R1049C	5’ GTCCAACGTGCGGTGCATGCACACAGC 3’	5’ GCTGTTGCATGCACCCACGTTGGAC 3’

Table S1. Oligonucleotide primers.

Primers used to create each KCC2 mutant using site-directed mutagenesis as described in the Experimental Procedures.

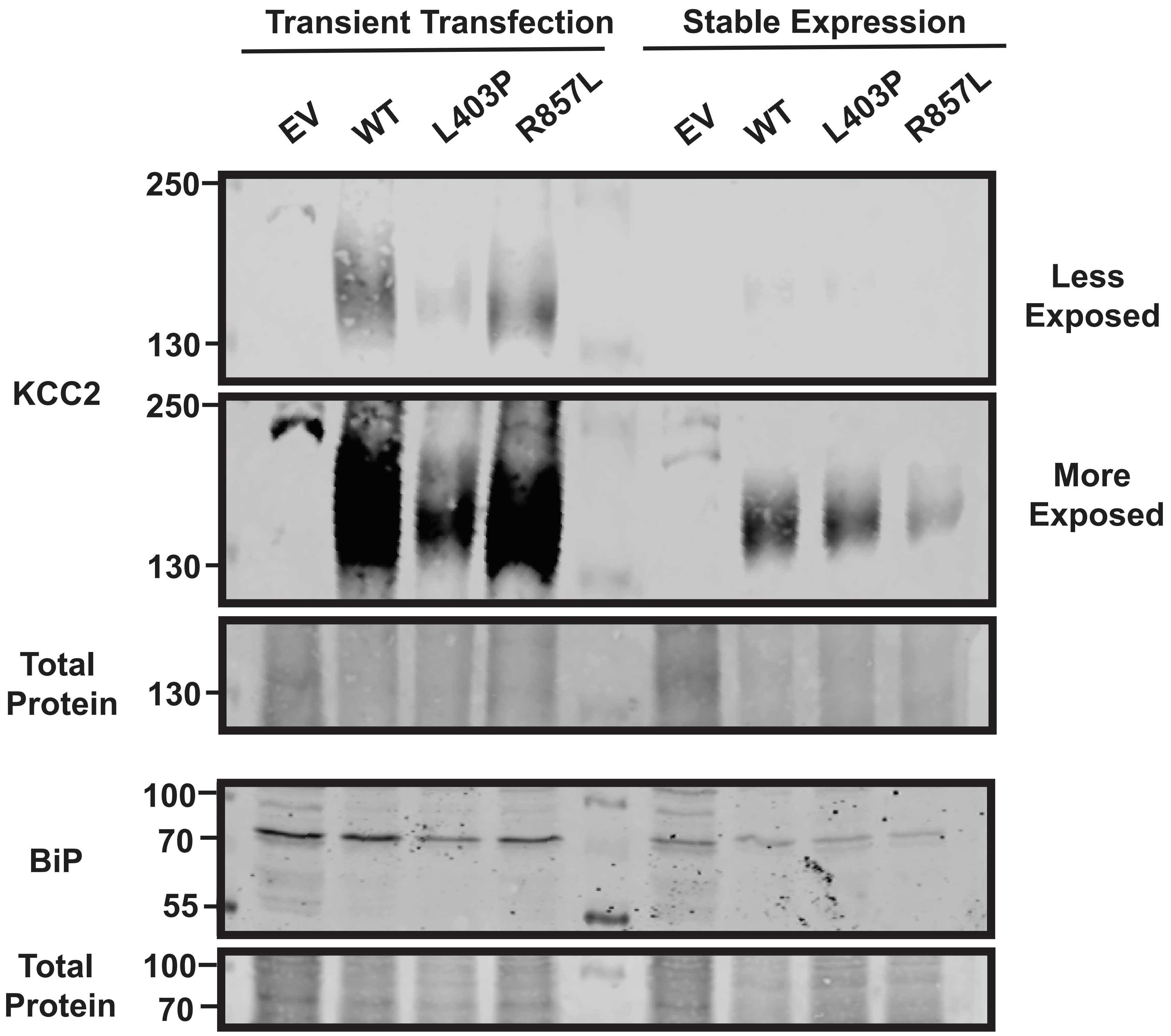

Figure S1. Relative KCC2 and BiP levels in transient or stable KCC2-expressing HEK293 cells.

Western blot analysis of HEK293 cells transiently or stably expressing WT, L403P or R857L KCC2. BiP was detected as an indicator of cell stress. Empty vector (EV) was used as a control, and total protein was used as a loading control.

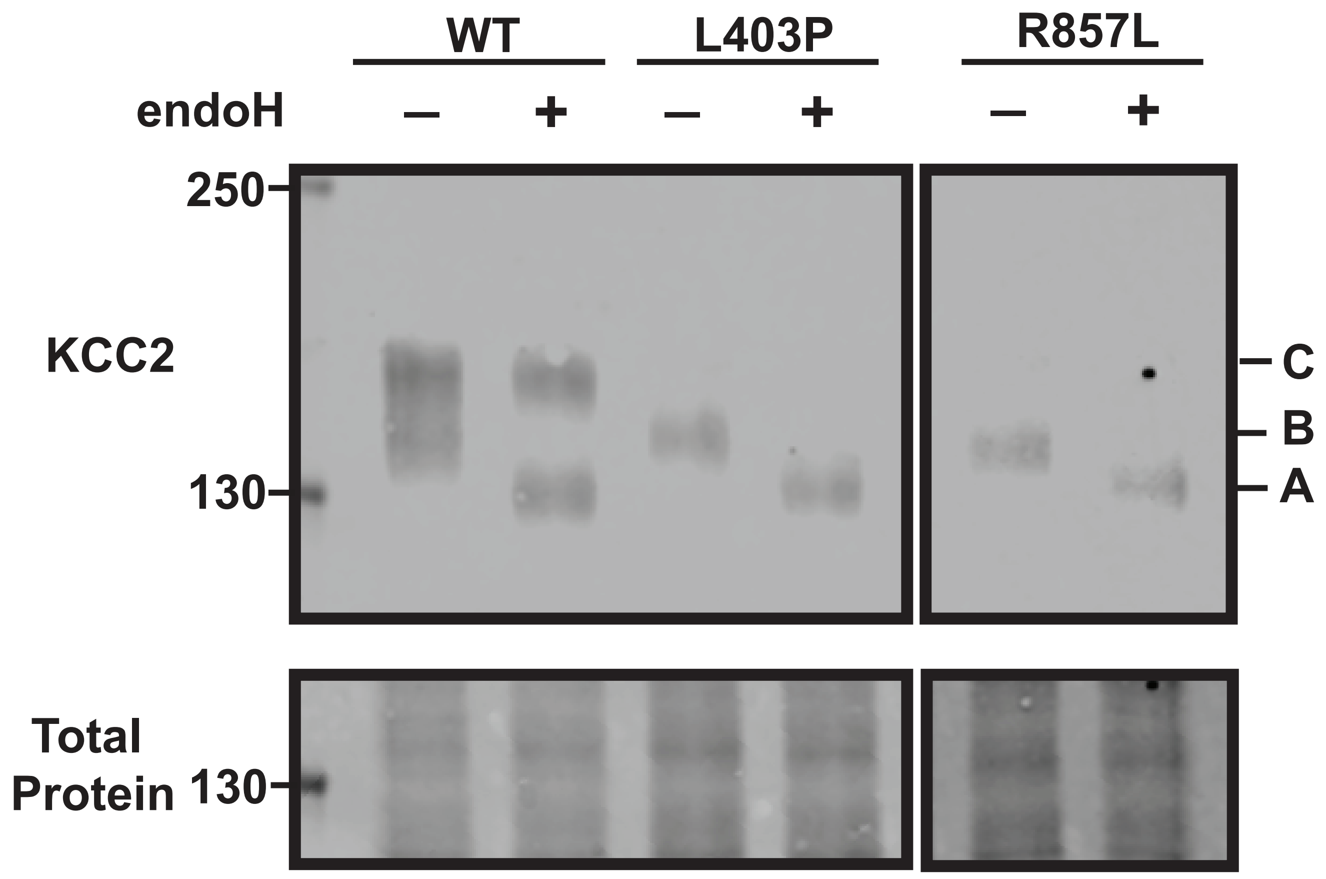

Figure S2. Detection of Golgi- and ER-modified glycans appended to KCC2 upon endoH treatment.

HEK293 cells stably expressing WT, L403P or R857L KCC2 were mock-treated or treated with endoH. Band C represents mature/Golgi-modified glycosylated protein, band B represents immature/ER-modified glycosylated protein, and band A represents unglycosylated protein. Total protein was used as a loading control.

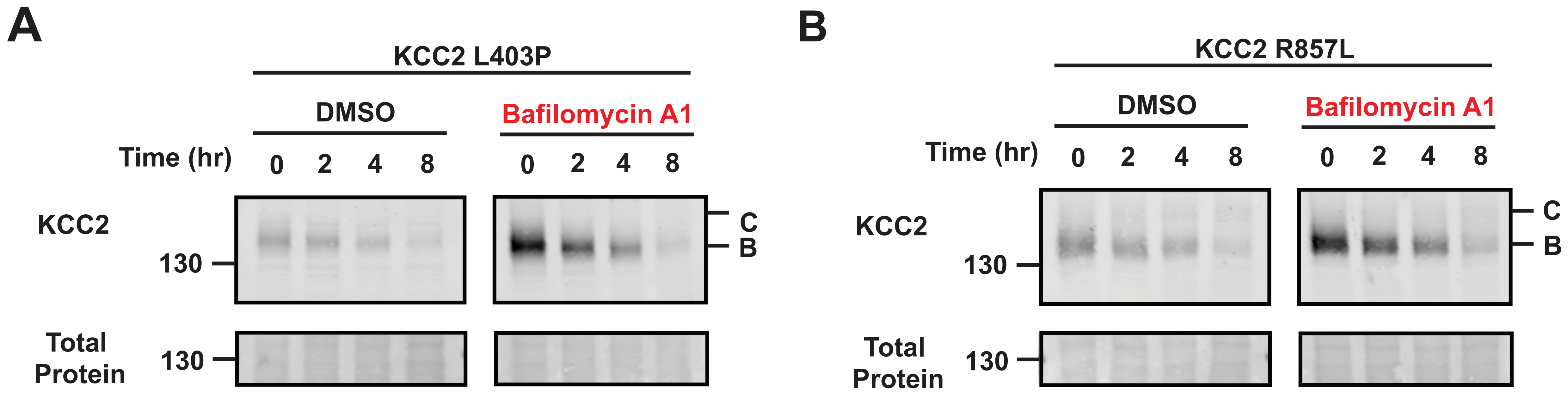

Figure S3. The amount of L403P and R857L KCC2 increases after bafilomycin A1 treatment of stably expressing HEK293 cells.

(A) Cycloheximide chase analysis of HEK293 cells stably expressing L403P KCC2. Cells were treated with bafilomycin A1 or vehicle control (DMSO). **(B)** Cycloheximide chase analysis of HEK293 cells stably expressing R857L KCC2. Cells were treated with bafilomycin A1 or vehicle control (DMSO). Total protein was used as a loading control.
